# Real time cardiac MRI: spline-based spatio-temporal reconstruction of spiral data

**DOI:** 10.1186/1532-429X-13-S1-P141

**Published:** 2011-02-02

**Authors:** Bénédicte MA Delattre, Jean-Noël Hyacinthe, Gunnar Krüger, Jean-Paul Vallée, Dimitri Van De Ville

**Affiliations:** 1 University of Geneva, Geneva University Hospital, Geneva, Switzerland; 2 Siemens Medical Solutions, Centre d'Imagerie Biomédicale (CIBM), Lausanne, Switzerland; 3 Ecole Polytechnique Fédérale de Lausanne (EPFL), University of Geneva, Lausanne and Geneva, Switzerland

## 

We propose a novel reconstruction method using a spline-based image model in both spatial and temporal dimensions that takes the advantage of the precise timing of each k-space sample to reconstruct image series at high time frames, independently from the original sampling rate of data and avoiding the temporal blurring that can affect other reconstruction methods like sliding window.

While MRI techniques have undergone considerable improvements since the early days, performing real time imaging is still challenging today. Despite the various original methods proposed until very recently, the usual way to reconstruct data at a higher frame rate is the sliding window technique that brings along intrinsic temporal blurring

The proposed image model is the following: *ρ*(*x,y,t*)*=Σ _n1,n2,n3_c_n1,n2,n3_β^1^*(*x-n_1_*)*β^1^*(*y-n_2_*)*β^α^*(*t-n_3_*) where *β^α^* is a spline function (α the spline degree). The spline model has the main advantage of allowing image reconstruction at arbitrary time points. In order to recover the Nyquist criteria, more data samples are included in the reconstruction by increasing the spline degree but they are weighted with the temporal information (zeroth degree neglects the temporal information of the samples as in nearest neighbor interpolation). The model was validated on an analytic phantom and then applied to real time data acquired from a healthy volunteer on a 3T scanner using a spiral sequence.

**Figure 1:**
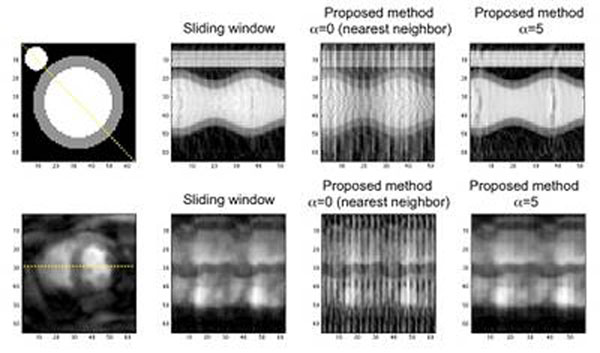
Upper, temporal profiles of sliding window (left) and proposed (right) reconstruction, for the numerical phantom; lower, for real-time data acquired on a healthy volunteer.

**Figure 2:**
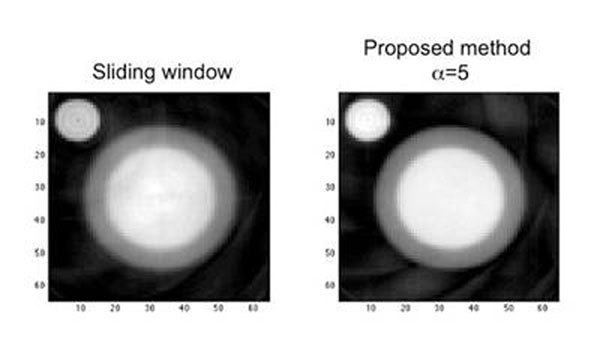
Illustration of blurring on sliding window reconstruction compared to proposed method for the analytic phantom.

Results obtained on the numerical phantom demonstrated that the time resolution of the reconstructed data could be improved by a factor of 3 when using a high enough spline degree, without sacrifying the SNR (11.5 dB for sliding window and 10.3 dB for the proposed method with α=5, whereas only 5.78 dB for α=0) (figure 1). Moreover, the edge strength of the endocardium was recovered for the proposed method (edge strength was 10.4e-2a.u. for sliding window and 13.9e-2a.u. for our method with α=5) (figure 2). The temporal profile obtained on real data with 5^th^ degree splines was comparable to the one obtained with the classical sliding window. Note that the number of spline coefficients (degrees of freedom of the model) remains fixed.

This novel approach is able to increase up to 3 times the temporal resolution of reconstructed images without introducing temporal blurring.

